# Supplementary material for: Efficient preservation and breakup of liquid sheets using screen-projected particles
Source: PLoS One. 2020 Feb 5;15(2):e0227590. doi: 10.1371/journal.pone.0227590 (PMC7001942; doi:10.1371/journal.pone.0227590)
Supplement: Attachment — Submitted filename: 2019_PLOS_One_Lee.pdf [file pone.0227590.s002.pdf]

Review of: Efficient Preservation and Breakup of Liquid Sheets Using Screen-Projected Particles

This article presents a method to improve the preservation and breakup of liquid sheets as well as improving the computational efficiency using a screen-projection method. The work adds novel ideas to previous publications. However, additional details need to be added.

1. One of the main contributions of this manuscript is the improved efficiency gained by the screen-projection of particles. It is not clear how the approach is similar or different from those in references [3 & 4]. Additional details should be provided to explicit state what the contribution is and how it compares to previous works. Furthermore, additional mathematical rigor is needed to describe what is solved in the screen-projected space and how. The fluid solver is stated to be based on reference [19] (page 10), however, this is a 3D solver and the manuscript states the "Navier-Stokes equation in 2D" (page 4) is solved.
2. The other main contribution is the addition of Eq. 11 to the avoid adding particles where the density is low and sheet breakup should occur. Additional discussion should be included to describe why this condition is different than modifying the constraints provied by Eq. 10. For example, increasing the value of $alpha_3$ in Eq. 10a would limit particle pairs that are close to each other and thus have a large density. Furthermore, the mathematical expression used to compute the density needs to be provided.
3. Corresponding author email address needs to be updated.
4. Page 2, line 42. Clarify that the level set method is used for "physically based simulations and CFD" *of interfaces*.
5. Page 3. Add a transition before line 93 that says something like: "A summary of the proposed algorithm is provided below:".
6. Page 4. The projection of the water particles from 3D to 2D space is performed using a "displacement function". This is confusing and the authors should consider using "projection function"
7. Page 4/5 The manuscript describes the "Extraction of Screened Water Particles". The idea of extraction seems confusing as particles are being selected but are not removed from the simulation. Consider modifying the description from "extracted"
8. The proposed method contains many constants that are not defined. Additional detail needs to be included to give values for the contants used for the results shown in the manuscript. Some of the variables include the projection matrix $\boldsymbol{P}$, ω, d_0, α_i for $i = 1, \ldots, 4$
9. Page 5, line 126-128. Details on how a single radius is computed from the radi in screen space. Provide the mathematical expression.

10. Page 5, line 141. It is unclear what backface particles are and additional detail should be provided.
11. Page 7, Fig. 5. The particles that have not been extracted are difficult to see as their color is the same as the solid. It is recommended to color these particles with a color that is different than the "thin particles".
12. Page 12, the results are described as "experimental", however all the presented results are simulation results with no experiments being performed. The language should be modified to be more explicit.

Grammatical Errors/Suggestions

1. Page 1, sentence on lines 3-5 is a fragment.
2. Page 2, sentence on lines 36-37 is awkward.
3. Page 3, line 60, change "improved this technique efficiently" to "improved the efficiency of this technique"
4. After many equations the next sentence is a continuation of the equation and should not be indented or captitalized. For example, see Eq. 3 which has "where" indented. Eq. 4 has the correct non-indented "where", but this where should be roman and not italicized.
5. Page 5, line 130. Add space between values and $(z_{i,j})$.
