# Supplementary material for: Efficient preservation and breakup of liquid sheets using screen-projected particles
Source: PLoS One. 2020 Feb 5;15(2):e0227590. doi: 10.1371/journal.pone.0227590 (PMC7001942; doi:10.1371/journal.pone.0227590)
Supplement: Attachment — Submitted filename: Revision_Letter.docx [file pone.0227590.s003.docx]

**Dear. Editors and Reviewers**

Thank you for spending your valuable time and expertise in thoroughly reviewing the article and providing thoughtful comments. Your comments really helped us improve the quality of our initial submission.

Thank you again for providing constructive suggestions how we could improve the paper.

Sincerely yours,

**Reviewer #1**

One of the main contributions of this manuscript is the improved efficiency gained by the screen-projection of particles. It is not clear how the approach is similar or different from those in references [3 & 4]. Additional details should be provided to explicit state what the contribution is and how it compares to previous works. Furthermore, additional mathematical rigor is needed to describe what is solved in the screen-projected space and how. The fluid solver is stated to be based on reference [19] (page 10), however, this is a 3D solver and the manuscript states the “Navier-Stokes equation in 2D” (page 4) is solved.

[Answer] References 3 and 4 are techniques for water rendering and foam simulation using a screen space approach. The difference with the proposed method is that only the water particles near the camera were selected using the depth away from the screen space to minimize the number of particles affecting the calculations during liquid sheet preservation. The shape of the particles was determined by assigning a radius to the particles, and in this process, the accuracy of the depth value varies depending on the size of the radius. Our paper does not calculate the exact depth map, but rather selects screened water particles, which yields sufficient computational efficiency in pruning unwanted particles. In addition, when projecting 3D water particles advected by 3D FLIP (Fluid-implicit particle) used as an underlying water solver to 2D screen space, only x and y information is projected. Since they have similar characteristics and motions to those obtained by solving the Navier-Stokes equation, they are approximated by selecting screened water particles. Of course, there are some differences from the exact Navier-stokes equation movement. (Page 13, line 343 in highlighted-manuscript file)

The other main contribution is the addition of Eq. 11 to the avoid adding particles where the density is low and sheet breakup should occur. Additional discussion should be included to describe why this condition is different than modifying the constraints provided by Eq. 10. For example, increasing the value of alpha3 in Eq.10a would limit particle pairs that are close to each other and thus have a large density. Furthermore, the mathematical expression used to compute the density needs to be provided.

[Answer] As you said, Eq. 10 is a formula that improves the condition for finding nearby particle pairs so that they can be connected better. As you can see, increasing $alpha_3 in Eq. 10a can result in fewer candidate particles because pairs that are in close proximity are ignored. However, this approach eventually affects Eqs. 10b and 10c, and as a result, too few candidate particles are likely to be selected. That's why we use conditions that work differently than Eq. 10. If $alpha_3 is too small, too complex liquid sheets will be created; if it is too large, liquid sheets will not be preserved. So we use Eq. 11 to represent the breakup of the liquid sheets using a variation of density rather than distance.

Corresponding author email address needs to be updated.

[Answer] Modifications have been made. (Page 1)

Page 2, line 42. Clarify that the level set method is used for "physically based simulations and CFD" of interfaces.

[Answer] Modifications have been made as follows. (Page 2, line 41 in highlighted-manuscript file)

Page 3. Add a transition before line 93 that says something like: "A summary of the proposed algorithm is provided below:".

[Answer] I agree with you and added that sentence. (Page 3, line 96 in highlighted-manuscript file)

Page 4. The projection of the water particles from 3D to 2D space is performed using a "displacement function". This is confusing and the authors should consider using "projection function"

[Answer] I agree with you. I changed the term "Displacement function" to "Projection function". (Page 4, line 110 in highlighted-manuscript file)

Page 4/5 The manuscript describes the "Extraction of Screened Water Particles". The idea of extraction seems confusing as particles are being selected but are not removed from the simulation. Consider modifying the description from "extracted"

[Answer] As I reviewed, I decided that “Selection” would be more appropriate, as you think, so I modified it. (Page 4, line 117 in highlighted-manuscript file)

The proposed method contains many constants that are not defined. Additional detail needs to be included to give values for the constants used for the results shown in the manuscript.

[Answer] I agree with you and add the parameters used in the paper to the table. (Page 14, Table 2 in highlighted-manuscript file)

Page 5, line 126-128. Details on how a single radius is computed from the radi in screen space. Provide the mathematical expression.

[Answer] The particle has three-dimensional coordinates of x, y, z, and we do the same for radius as we compute the projected screen coordinates x_p, y_p, z_p by applying Eq.2 to these three-dimensional coordinates. In 3D, the radius of the X, Y, and Z axes is the same, and since r_x and r_y have the same value after the projection, any of r_x and r_y can be used as the value of r_p. (Page 7, line 132 in highlighted-manuscript file)

Page 5, line 141. It is unclear what backface particles are and additional detail should be provided.

[Answer] Back-face particles are a term for particles that are not visible from the camera. In the field of computer graphics, the technique of not rendering an invisible face from the camera is called “back-face culling,” and the term is used in this regard. To help the reader understand, I added a sentence to explain this in more detail before using the word “Back-face” as follows. (Page 5, line 149 in highlighted-manuscript file)

Page 7, Fig. 5. The particles that have not been extracted are difficult to see as their color is the same as the solid. It is recommended to color these particles with a color that is different than the "thin particles".

[Answer] Modifications have been made. (Page 7, Fig 5 in highlighted-manuscript file)

Page 12, the results are described as "experimental", however all the presented results are simulation results with no experiments being performed. The language should be modified to be more explicit.

[Answer] As I reviewed, I decided that “Simulation” would be more appropriate, as you think, so I changed the term "Experimental result" to "Simulated result". (Page 12 in highlighted-manuscript file)

Grammatical Errors/Suggestions

- Page 1, sentence on lines 3-5 is a fragment.
- Page 2, sentence on lines 36-37 is awkward.
- Page 3, line 60, change "improved this technique efficiently" to "improved the efficiency of this technique".
- After many equations the next sentence is a continuation of the equation and should not be indented or captitalized. For example, see Eq. 3 which has "where" indented. Eq. 4 has the correct non-indented "where", but this where should be roman and not italicized.

[Answer] I agree with you and modifications have been made.

Page 5, line 130. Add space between values and (z_i,j ).

[Answer] I agree with you and modifications have been made. (Page 5, line 138 in highlighted-manuscript file)

**Reviewer #2**

Using full 3d particles to select candidates vs. screen particles to select candidates, to see difference in visual fidelity.

[Answer] There is a clear difference between the results using screened particles and the results using whole 3D particles. The visual quality seen at a given viewpoint does not differ significantly between the two methods because the liquid sheets are represented in the area seen on the screen from the camera. Sudden appearance of particles in invisible space from the camera can affect the quality of the liquid sheets because they are not selected as screened particles in the previous frame. However, once a particle is selected as a screened particle, the liquid sheet preservation process is applied, so it is not completely excluded from the later liquid sheet generation process. Our experiments in various scenes confirm that the liquid sheets are robustly represented in the various scenes.

How the algorithm works for a rapid camera motions, would be it be robust without exhibiting abrupt and non-natural artifacts on the thin sheet constructions.

[Answer] As answered in the previous question, the proposed study can represent liquid sheets stably even when the camera is moving. When the camera moves sharply, the moving speed of the camera should be considered. For example, assuming that the camera rotates in a circle, if the rotation angle per frame is large, particles in other regions will be selected as screened particles every frame, and continuity between frames may not be guaranteed. As can be seen in the attached result video, almost no noticeable artifacts are found because almost identical particles are selected as screened particles between successive frames unless the camera moves too fast. The rotation angle of the camera used in the paper was set to 30 degrees per frame.
